# Health-Related Quality of Life in Patients with Hepatocellular Carcinoma Post-Surgery: A Scoping Review

**DOI:** 10.7150/ijms.115946

**Published:** 2025-07-28

**Authors:** Wei-Zheng Zhang, Kok-Yong Chin, Roshaya Zakaria, Nor Haty Hassan

**Affiliations:** 1 Department of Nursing, Faculty of Medicine, Universiti Kebangsaan Malaysia, Malaysia.; 2 Department of Pharmacology, Faculty of Medicine, Universiti Kebangsaan Malaysia, Malaysia.

**Keywords:** hepatocellular carcinoma, health-related quality of life, surgery, liver resec-tion, liver transplantation, post-operative care, scoping review

## Abstract

**Background**: Hepatocellular carcinoma (HCC) is a leading cause of cancer-related mortality worldwide. While surgical treatments, including liver resection and transplantation, offer curative potential, they significantly impact patients' health-related quality of life (HRQoL). This scoping review aims to comprehensively map the existing literature on HRQoL following surgical treatment for HCC by identifying commonly used assessment tools, variations across surgical approaches, and key influencing factors.

**Methods**: A comprehensive literature search was conducted in PubMed, Scopus, and Web of Science from inception to February 2025, adhering to the Joanna Briggs Institute (JBI) Scoping Review methodology and the PRISMA-ScR checklist. Studies were included if they assessed post-operative HRQoL in HCC patients using validated instruments. Two independent reviewers screened, selected, and extracted data from eligible studies.

**Results**: Of the 1,275 articles retrieved, 13 met the inclusion criteria. Sample sizes ranged from 66 to 332, with studies included conducted in the USA, China, Germany, Spain, Egypt, and Japan. The most frequently used HRQoL assessment tools were SF-36, FACT-Hep, and EORTC QLQ-C30. Findings revealed an initial decline in HRQoL postoperatively, followed by gradual recovery over 3-12 months. Liver transplantation generally resulted in substantial long-term HRQoL compared to liver resection, although challenges associated with immunosuppressive therapy persisted. Key factors influencing HRQoL included preoperative depression, post-operative complications, disease recurrence, and socioeconomic variables such as age, gender, and family support.

**Conclusions**: Post-operative HRQoL in HCC patients follows a dynamic trajectory, which emphasizes the need for patient-centered care strategies. Standardized HRQoL assessments and longitudinal studies are crucial for enabling cross-study comparisons and guiding targeted interventions to optimize recovery and long-term well-being.

## 1. Introduction

Hepatocellular carcinoma (HCC) is the most prevalent primary liver cancer and a significant global health problem. Globally, it accounts for over 900,000 new cases and approximately 830,000 deaths annually, making it the third leading cause of cancer-related death 1. Liver resection and transplantation are considered first-line curative treatments for patients with early HCC 2. While these interventions may extend survival, the post-operative period is often characterized by considerable physical, psychological, and social burdens that can have a substantial impact on a patient's health-related quality of life (HRQoL) 3. With increasing emphasis on patient-centered care, understanding the long-term effect of surgery on HRQoL is essential to optimize post-operative care and guide supportive care interventions 4.

HRQoL is a multifaceted construct encompassing physical, psychological, and social functioning 5. For patients afflicted with HCC, their HRQoL is influenced not only by post-operative recovery and surgical trauma but also by the psychological impact of cancer diagnosis and treatment, risk of recurrence, and late complications 6. Various kinds of instruments have been applied to assess HRQoL, ranging from general instruments like the 36-Item Short Form Survey (SF-36) to condition-specific instruments like the Functional Assessment of Cancer Therapy-Hepatobiliary (FACT-Hep) and European Organisation for Research and Treatment of Cancer Quality of Life Questionnaire Core 30 (EORTC QLQ-C30) 7-9. These measures of assessment acquire critical data on patient's subjective perception of well-being and functioning, making it possible to plan proper clinical and supportive care interventions.

Despite the growing list of research findings on HRQoL of HCC patients after surgery 10-12, no comprehensive scoping review has synthesized available evidence. This is mostly because earlier reviews, while concentrating mainly on survival and perioperative complications, have neglected the worldwide implications of procedures on general health 13. In addition, differing studies vary broadly in measuring HRQoL in terms of surgical modalities, measuring instruments, and predictors 13-15. Given the heterogeneity of studies in the literature and in accordance with guidelines 16, a scoping review was undertaken to map existing research, identifying the types of evidence available and research gaps carefully. Therefore, this review aimed to synthesize the most recent evidence pertaining to HRQoL in HCC patients post-surgery.

## 2. Materials and Methods

This scoping review was conducted following the Joanna Briggs Institute (JBI) framework and adhered to the Preferred Reporting Items for Systematic Reviews and Meta-Analyses extension for Scoping Reviews (PRISMA-ScR) checklist 17. The PRISMA-ScR checklist is available in Supplementary Material 1. A review protocol was entered into the Open Science Framework (Registered DOI: https://doi.org/10.17605/OSF.IO/UA2HD).

### 2.1. Identifying the Research Question

A comprehensive literature review was conducted to explore HRQoL in patients with HCC following surgical intervention. The research questions that directed the scoping review were as follows:

- What are the differences in HRQoL associated with different surgical procedures?
- What instruments have been used to assess HRQoL in patients diagnosed with HCC?
- What are the factors influencing the HRQoL in patients with hepatocellular carcinoma after surgery?

### 2.2. Identifying the Relevant Studies

This scoping review included studies published in peer-reviewed journals. A search was conducted in PubMed, Scopus, and Web of Science from inception to February 2025 to identify the HRQoL in patients with HCC following surgical intervention. As no prior scoping review on this topic was identified, no publication date restrictions were applied. The full search strings for each database are provided in Supplementary Material 2. Studies were included if they met the following criteria: (1) patients with HCC without metastasis of other sites; (2) patients after-surgery related to liver cancer; (3) any types of surgery; (4) related to quality of life after surgery; (5) utilize at least one questionnaire or tool to evaluate quality of life; (6) primary studies; (7) published in English; (8) publication years up to the date of search. The exclusion criteria were: (1) review, protocol, case report, expert consensus, conference abstracts and proceedings, thesis, and dissertations; (2) inability to obtain full text despite attempts to contact the authors and search through institutional resources.

### 2.3. Study Selections

**Figure 1:**
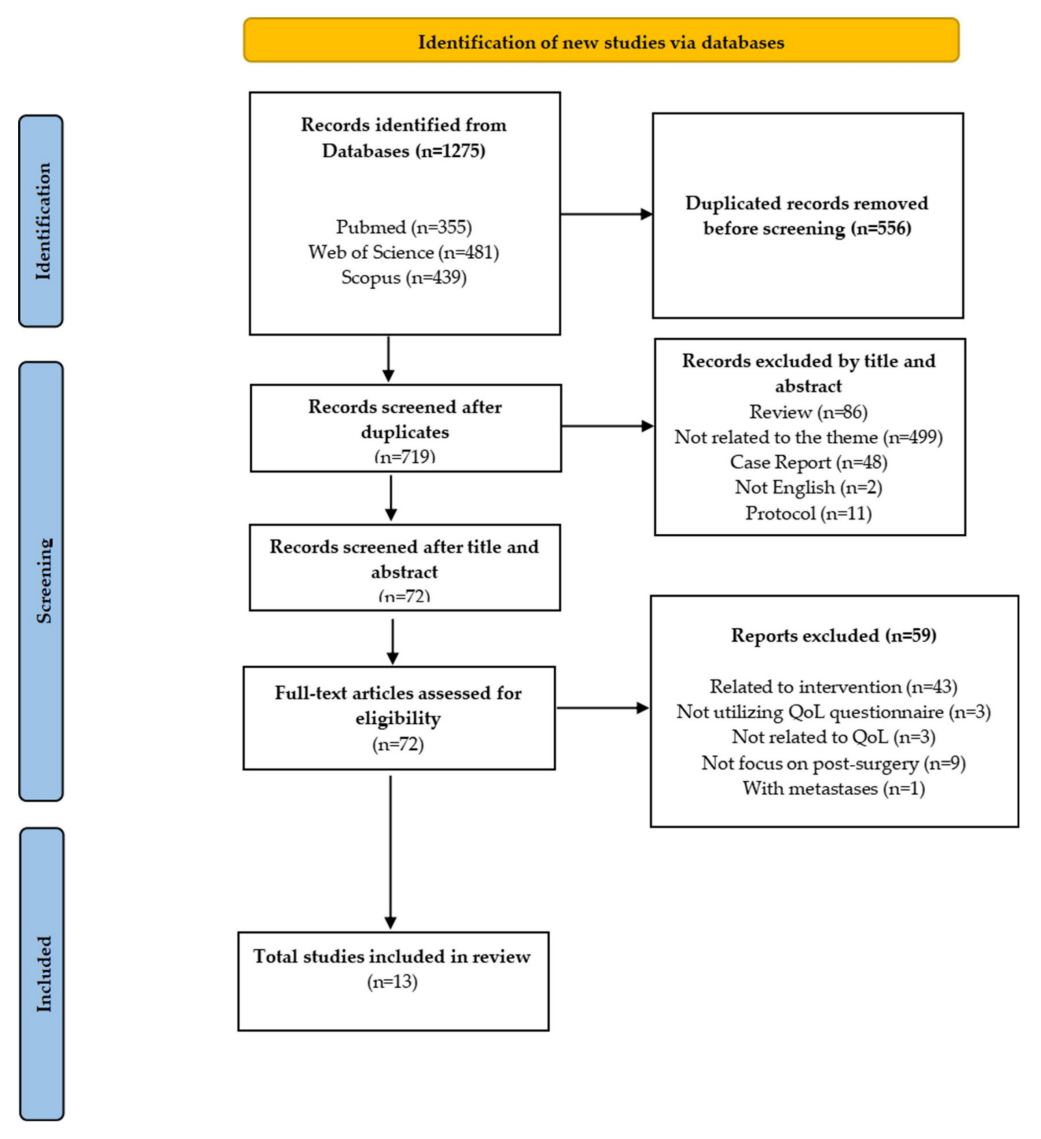
Flowchart of PRISMA applying to the study selection process.

Two independent researchers (WZZ and KYC) screened titles and abstracts, followed by a full-text review based on the eligibility criteria. Discrepancies in study selection were addressed through discussion. Figure 1 provides a comprehensive overview of the article screening and selection process. The overview of the screening and article selection process is shown in Figure 1.

### 2.4. Charting Data

A standardized data extraction form was used to collect key study characteristics, including the primary author's name, publication year, country of study, study objectives, study design, participant characteristics and sample size, type of surgery, HRQoL assessment methods, and major findings. Data extraction was conducted independently by both researchers.

### 2.5. Collating, Summarizing, and Reporting Results

Extracted data were compared and synthesized into a single dataset for analysis. The results were presented in tables that included comprehensive descriptions of study characteristics and the patients' HRQoL levels.

## 3. Results

### 3.1. Characteristics of Included Studies

This review analyzed the recent HRQoL outcomes in patients with HCC following surgical intervention. As shown in Figure 1, the initial search identified 1,275 articles, of which 719 remained after duplicate removal. After screening titles and abstracts, 72 full-text articles were assessed for eligibility. After applying the inclusion and exclusion criteria, 13 articles were identified as relevant and included in this scoping review.

**Figure 2:**
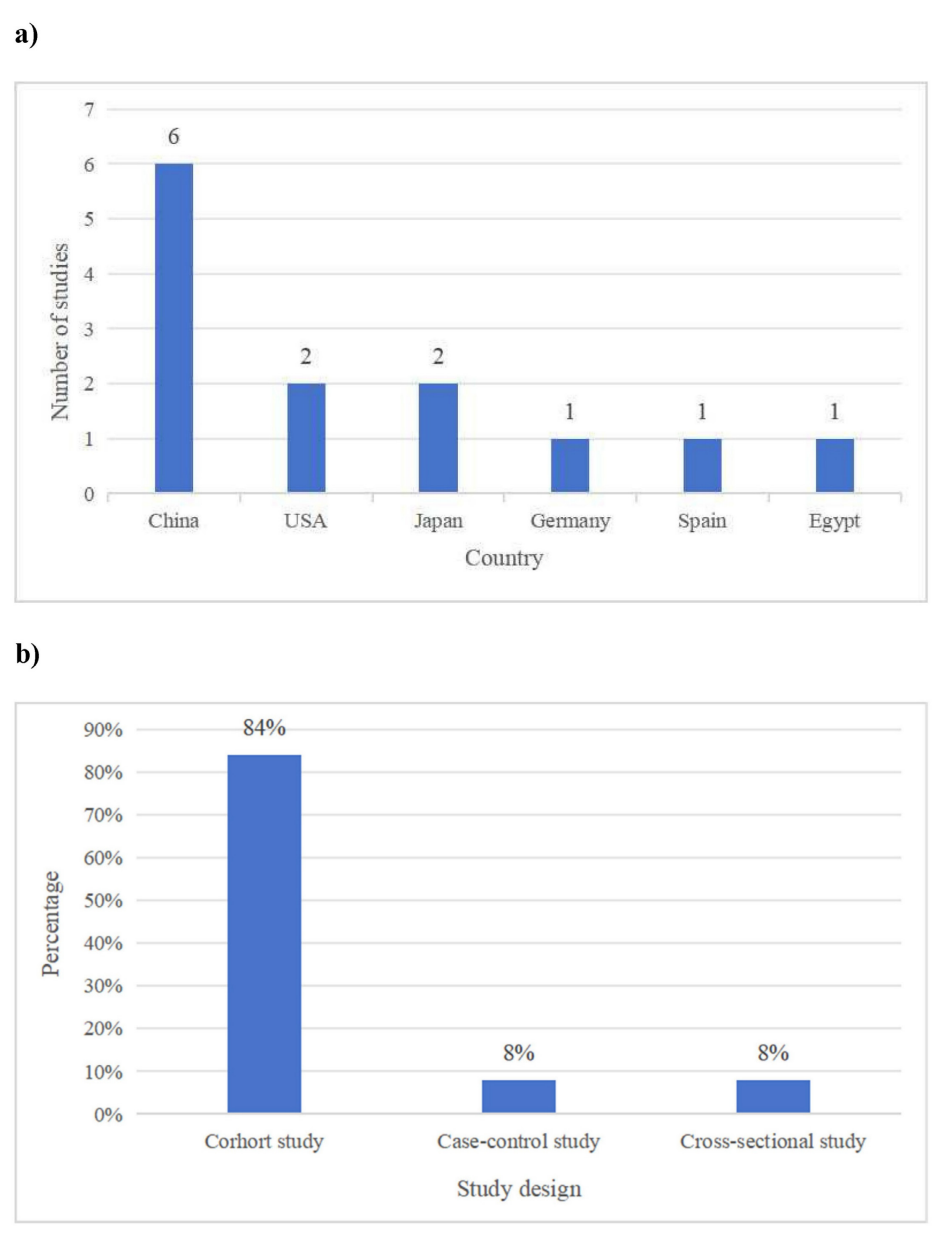
** a)** The distribution of studies by country. **b)** The distribution of studies by study design

Thirteen studies met the inclusion criteria, with sample sizes ranging from 66 to 332. The studies were conducted in various countries, including the USA, China, Germany, Spain, Egypt and Japan. The distribution of studies by country is illustrated in Figure 2. The majority were cohort studies (n=11), while the others were case-control (n=1) and cross-sectional studies (n=1). The distribution of studies by study design is provided in Figure 2.

The baseline demographic characteristics of included studies are summarized in Table 1. Most studies reported ages ranging between 50 and 65 years. Male predominance was consistent across studies, with male participants constituting approximately 60%-80% of the cohorts. Commonly reported aetiologies included hepatitis B virus (HBV), hepatitis C virus (HCV), and alcohol-related liver disease. Liver cancer staging primarily utilized Barcelona Clinic Liver Cancer (BCLC) or Tumor, Node, Metastasis (TNM) classifications, with most participants classified at early or intermediate stages.

### 3.2. HRQoL Trend in Patients with HCC Post-Surgery

A summary of varying trends in HRQoL among patients is reported in Table 2. The studies indicated varying trends in HRQoL among patients undergoing surgical treatment for HCC. Several studies observed an initial post-operative decline in HRQoL, followed by gradual recovery. Tohme et al.^18^ found that HRQoL decreased at 4 months post-surgery but returned to baseline levels by 8 months and remained stable at 12 months. Similarly, Chen et al.^22^ reported that HRQoL declined significantly within 2-10 weeks after surgery but recovered to preoperative levels by 4 months, with further improvements in long-term survivors. Chiu et al.^19^ noted a significant improvement in HRQoL by 3 months post-surgery, plateauing at 6 months. In contrast, Extraviz et al.^23^ and Mabrouk et al.^27^ reported significant improvements in HRQoL after liver transplantation, particularly among patients with severe preoperative disease.

### 3.3. Surgical Types and Follow-up Duration

In Table 3, a brief overview indicating various surgical procedures and how long follow-up periods typically last is included. The different kinds of surgeries looked at include liver resection, which was noted for its seven instances in studies 18, 19, 22, 23, 25, 29, 30, and liver transplantation, which appeared twice 27, 28. Several research evaluated multiple surgical methods. Feldbrugge et al. 20 compared laparoscopic liver resection with orthotopic liver transplantation, allowing some examination of outcomes related to HRQoL in these procedures. Lei et al. 21 observed both liver transplantation and liver resection to better understand how HRQoL could differ between these two surgical options. Xie et al. 24 did a comparative study on HRQoL outcomes, studying liver resection alongside transarterial chemoembolization (TACE). Chie et al. 26 provided a thorough investigation in China, contrasting HRQoL outcomes of liver resection, ablation, and embolization. Follow-up times varied significantly, ranging from as short as three months to as long as five years. It is important to note that longitudinal studies frequently reported several follow-up time points, including those at six months, twelve months, and twenty-four months.

### 3.4. HRQoL Assessment Tools

Table 4 provides a summary detailing the tools used to assess HRQoL in those studies considered. The Short Form-36 Health Survey (SF-36) was the most utilized HRQoL assessment tool with its seven occurrences 19-21, 23, 24, 27, 29. Following that, the second commonly employed instrument was the Functional Assessment of Cancer Therapy-Hepatobiliary (FACT-Hep), used in two studies 18, 30. There were also instances of the Gastrointestinal Quality of Life Index (GQLI) being utilized once 22, the WHOQOL-BREF appeared one time 23, and the EORTC QLQ-C30 tool was noted one time 24. Moreover, one study applied a self-designed validated questionnaire, as noted by Ueno et al. 25.

### 3.5. Differences in HRQoL Among Types of Surgery

A summary of HRQoL outcomes in connection to various surgical methods for HCC is located in Table 5. It can be seen that HRQoL results tended to differ among the surgical techniques, and distinct recovery patterns were noticed in patients undergoing liver resection, liver transplantation, and minimally invasive procedures. It appears that liver resection had an initial decline in HRQoL after surgery; however, there was a gradual return to better levels. Studies by Chiu et al.^18^, ^19^ and Tohme et al.^18^ pointed out that HRQoL might improve significantly within a timeframe of 3 to 12 months. Nevertheless, the long-term outcomes seemed to be shaped by factors that include tumor recurrence. It was generally reported that liver transplantation led to better HRQoL, as was indicated by Mabrouk et al.^27^, but problems related to immunosuppressive therapy and complications post-transplant could negatively influence long-term well-being. In addition, minimally invasive techniques, which include laparoscopic liver resection, showed HRQoL results that were similar to what was seen in open surgery and transplantation, as reported by Feldbrugge et al. 20; still, details on long-term outcomes remain uncertain. Comparative studies, like those from Lei et al.^21^, suggested that long-term HRQoL outcomes might not differ greatly between liver resection and transplantation, although liver resection was noted to possibly lead to better HRQoL than non-surgical methods such as TACE.

### 3.6. Factors Influencing HRQoL in HCC Patients

Table 6 presents an overview of major factors that had an impact on HRQoL outcomes in patients with HCC before and after undergoing surgery. Various factors, both prior to surgery and after, were found to affect the HRQoL outcomes. Tohme et al. 18 noticed that preoperative depressive symptoms, pain, and fatigue were key contributors to a decline in HRQoL. On the other hand, post-operative aspects, which included disease recurrence, tumor features, and ongoing symptoms, seemed to similarly influence long-term HRQoL. Socioeconomic and demographic factors such as age and gender, as well as education level and family support, were pointed out by Chiu et al. 19 and Lee et al. 28. Factors related to the surgery itself, for instance, the extent of the surgery, as highlighted by Chen et al. 22, post-operative issues, and various complications, which were noted by Feldbrugge et al. 20 and Mabrouk et al. 27 also markedly influenced HRQoL outcomes. The recurrence of HCC was consistently identified as a significant factor that led to a decline in HRQoL over time, as seen in three studies 24, 25, 30.

### 3.7. Changes in HRQoL Over Time

Table 7 illustrates the changes over time regarding HRQoL after surgery that was done for HCC. Generally, HRQoL appeared to drop initially right after surgery, and then there was a slow process of recovery afterward, with, in some cases, improvements that were sustained over an extended period. Some studies, such as Mise et al. 29, discovered that physical health issues reached their worst point around three months following the surgery but later returned to what it used to be by around six months. Meanwhile, mental health reportedly went beyond what it was before the surgery. Other studies variously noted, such as those by Chiu et al. 19 and Poon et al. 30, showed that there were significant gains in HRQoL after surgery, with benefits that tended to last, especially in patients who did not experience any recurrence. However, Extraviz et al. 23 reported that although liver transplantation significantly improved HRQoL, most patients' HRQoL scores remained below general population norms at long-term follow-up.

## 4. Discussion

This scoping review mapped the available evidence on HRQoL in patients with HCC after surgery. The findings highlight significant variations in HRQoL outcomes across different surgical approaches, assessment tools, and influencing factors. Understanding these patterns is essential for improving post-operative care and guiding future research.

### 4.1. HRQoL in Patients with HCC After Surgery

The results indicate that HRQoL in HCC patients generally follows a trajectory of initial decline post-surgery, with gradual recovery over time 18, 21, 22, 24. Multiple studies reported a temporary deterioration in HRQoL within the first few months after surgery 18, 19, 22, attributed to surgical trauma, post-operative complications, and emotional distress. This observation aligns with the broader literature on post-operative resilience in oncological populations, where functional recovery often correlates with physiological adaptation and psychosocial adjustment to survivorship 6, 31-33. However, the extent and duration of HRQoL recovery varied significantly across studies, depending on factors such as surgical technique, baseline health status, and recurrence risk.

Notably, this overall trajectory may not be uniform across all HRQoL domains. A closer look at domain-specific outcomes in the included studies suggests that physical functioning and role limitations tend to show earlier improvement following surgery, while emotional and social domains may recover more gradually or remain impaired. For instance, several studies reported rapid recovery in physical functioning within 3-6 months, whereas mental health and social participation scores lagged behind or remained below baseline levels 22, 24, 27. These domain-level variations highlight the importance of designing tailored interventions that address both physical rehabilitation and psychosocial support throughout the post-operative recovery period.

In addition to surgical variables, patient-specific baseline characteristics may also shape HRQoL trajectories. Baseline characteristics varied across the included studies and may partly explain differences in HRQoL outcomes. Most studies 18, 19, 27 included predominantly male patients, with mean ages ranging from the mid-40s to early 60s. The study 21 with younger cohorts tended to report better recovery in physical domains. HBV was the most common etiology, while some included HCV and alcohol-related disease. Differences in etiology may influence HRQoL via recurrence risk or treatment effects. Several studies included patients with early-stage HCC, which is often associated with better outcomes. In many cases, HRQoL returned to baseline levels or improved with time, particularly in patients without disease recurrence 20, 27. These demographic and clinical variations highlight the importance of accounting for baseline factors when interpreting HRQoL trajectories and tailoring supportive care.

Minimally invasive techniques, such as laparoscopic resection, demonstrated comparable or even superior short-term HRQoL outcomes compared to open surgery, with advantages in reduced post-operative pain and faster recovery times 20. Liver transplantation, while associated with sustained HRQoL improvements in patients with advanced disease, revealed that despite resolving cirrhosis-related symptoms, recipients often scored below population norms in mental health domains, likely due to immunosuppression burdens and existential concerns 34-36.

Current literature suggests that some interventions, especially targeted interventions, are essential to improve post-surgery HCC patients' HRQoL. For example, multidisciplinary treatment approaches, particularly an individualized approach, can significantly manage the HRQoL in HCC patients post-surgery by considering liver function, cancer stage, and patient preferences 37. Moreover, psychological interventions can effectively improve their HRQoL by reducing patient's emotional distress. Significant improvements in psychological distress and sleep quality were observed in a randomized controlled trial examining guided self-help mindfulness-based therapies in patients with HCC 38. In addition, palliative treatment has also been demonstrated to reduce psychological and physical symptoms and increase survival for cancer patients 39. Thus, increasing access to palliative care and enhancing training and resources for medical professionals can assist in maximizing HRQoL in patients with HCC after surgery, particularly in the later stages of the illness.

### 4.2. HRQoL Assessment and Measurement Tools

A key finding of this review is the diversity of instruments used to measure HRQoL, including both generic (e.g., SF-36) and disease-specific tools (e.g., FACT-Hep, EORTC QLQ-C30). SF-36 was utilized most frequently, most likely due to its high relevance in assessing general health status 40-42. Disease-specific instruments such as FACT-Hep and EORTC QLQ-C30 can provide more sensitive measurements of cancer symptoms and treatment effects 7, 43-45. While they have advantages, they differ in sensitivity and scope, with SF-36 being more suitable for broad comparisons and FACT-Hep providing symptom-specific data better 46.

A recent systematic review 47 has highlighted the diversity in HRQoL instruments used in studies on HCC patients undergoing systemic therapies. The review found that eight different HRQoL instruments were employed across studies, including SF-36, EQ-5D (EuroQol 5-Dimensions), FACT-G, QLQ-C30, QlQ-HCC18 (European Organisation for Research and Treatment of Cancer Quality of Life Questionnaire-Hepatocellular Carcinoma Module), FACT-Hep (Functional Assessment of Cancer Therapy-Hepatobiliary), FACT-HS (Functional Assessment of Cancer Therapy-Hepatocellular Symptoms), FHSI-8 (Functional Assessment of Cancer Therapy-Hepatobiliary Symptom Index-8). This diversity, while offering flexibility in assessing HRQoL, also makes it challenging to compare results and establish clear standards for evaluation. The use of multiple instruments with varying levels of sensitivity can lead to inconsistency in measuring treatment outcomes across studies.

To enhance homogeneity, subsequent studies should accord high priority to the utilization of validated instruments, such as the EORTC QLQ-C30 and FACT-Hep, which are more sensitive to cancer symptom-related effects 48. Additionally, the utilization of both generic and disease-specific tools may provide a better reflection of the overall health and treatment effects of the patients 49. Consensus guidelines to measure HRQoL would become easier to establish with formation, as they would standardize time points and key domains. Multicenter trials of large size with standardized measurement protocols are needed to derive strong evidence and enable good-quality meta-analyses.

### 4.3. Differences in HRQoL Among Surgical Approaches

A comparison of the different surgical procedures revealed distinct recovery patterns. The most common operation, liver resection, was characterized by an initial post-operative worsening of HRQoL, followed by progressive improvement during subsequent months 18, 21, 22, 24. In contrast, liver transplantation often resulted in significant long-term improvement in HRQoL, particularly in patients with advanced preoperative disease 23. A systematic review by Yang et al. 50 further confirmed that liver transplantation leads to substantial long-term improvements in HRQoL, particularly in functional domains such as mobility and self-care, compared to preoperative status. This aligns with other studies that suggest that liver transplantation can restore patients' HRQoL to levels comparable with the general population, although physical function remains worse. However, patients transplanted for HCC may experience persistent psychological distress related to cancer recurrence and long-term immunosuppression, which may differentiate their mental health outcomes from those transplanted for non-malignant indications.

Interestingly, trials of minimally invasive surgery, such as laparoscopic liver resection, reported similar or even superior HRQoL results compared to open surgery, though long-term evidence remains limited 20. These findings emphasize the importance of selecting the most appropriate surgical approach based on the patient's profile and projected HRQoL trajectory.

In addition to the surgical approach, a variety of supplementary techniques have shown promise in improving post-surgery HRQoL in HCC patients. Higher adherence to enhanced recovery after surgery (ERAS) procedures has been linked to significantly lower post-operative complication rates and shorter hospital stays 51. Furthermore, a recent randomized controlled trial 52 demonstrated that a digital rehabilitation program significantly increased post-operative exercise adherence and cardiovascular endurance in HCC patients, underscoring the positive impact of digital interventions on their HRQoL.

It is important to note that many of the included studies were conducted prior to the widespread adoption of laparoscopic and robotic liver surgery, as well as the implementation of ERAS protocols. These modern techniques have been shown to reduce post-operative complications, shorten hospital stays, and potentially improve HRQoL outcomes. As such, the HRQoL data presented in this review may not fully reflect the benefits associated with current surgical advancements. There is an urgent need for contemporary studies that evaluate HRQoL in the context of minimally invasive approaches and standardized perioperative care protocols.

### 4.4. Factors Influencing HRQoL

This review revealed several factors in post-operative HRQoL results in HCC patients. Preoperative factors, i.e., preoperative physical status, psychological status, and economic and social conditions, were mainly significant for predetermining HRQoL following surgery.

Particularly, preoperative depression, fatigue, and pain were indicated by lower HRQoL scores 18. According to studies 31, 48.70% of HCC patients experienced differential levels of depression. Living depression may adversely impair the HRQoL of HCC patients. Additionally, fatigue is a frequent and disabling symptom among patients with liver cancer, with a prevalence of up to 90% 53. The disorder is generally the consequence of the gradual loss of energy reserves during illness 54. Research has established that fatigue among patients with liver cancer results from a variety of factors, ranging from tumor burden and treatment toxicities to pre-existing liver dysfunction 53. This chronic fatigue extensively influences patients' HRQoL and compromises their physical function, emotional status, and activities of daily life. Pain is another common and significant symptom in HCC patients, present in as many as 90% of them due to tumor overgrowth, invasion of overlying tissue, and compression of the nociceptors of adjacent organs 55. Pain of moderate to severe grade is present in most patients. This condition aggravates physical pain and substantially reduces their HRQoL 55. In brief, optimal pain management is important in improving global well-being and therapeutic outcomes among such patients.

Post-operative considerations, including surgical complications, recurrence of the disease, and side effects due to treatment, also significantly contributed to HRQoL outcomes 22, 27. In contrast to surgical patients, those receiving systemic therapies for advanced HCC often experience significant treatment-related toxicities that adversely affect HRQoL. In contrast to surgical patients, those receiving systemic therapies for advanced HCC often experience significant treatment-related toxicities that adversely affect HRQoL. As reported in a recent systematic review 47, common adverse effects such as hand-foot syndrome, fatigue, hypertension, and diarrhea are prevalent in patients receiving tyrosine kinase inhibitors or immunotherapy. These side effects, largely absent in surgical patients, contribute to early declines in physical and emotional well-being, underscoring the need for individualized symptom management depending on treatment modality. This result is in line with earlier research, which has also reported significantly lower physical, functional, and emotional HRQoL in HCC patients compared to the general population because of disease-related complications and treatment-related adverse events 3.

Demographic factors like age, gender, level of education, and family support also proved to be significant predictors of HRQoL 19, 28. This association may be accounted for by worse recovery potential after post-operative complications or disease recurrence in older patients, which adds to a greater impact on HRQoL 56. Furthermore, higher education may be associated with higher health awareness, better self-management skills, and good family support as a protective factor, reversing the ill effects due to recurrence or complications 57, 58.

Another important yet underexplored factor influencing post-surgical HRQoL is the use of adjuvant systemic therapies. Chemotherapy, targeted therapies (e.g., sorafenib, lenvatinib), and immunotherapies are increasingly integrated into HCC treatment pathways. These therapies are known to have substantial side effect profiles, including fatigue, gastrointestinal symptoms, and hand-foot syndrome, that may significantly impact HRQoL 47. However, most of the included studies did not clearly report or stratify patients based on exposure to systemic treatments, limiting the ability to isolate their effects. This represents a critical confounder and highlights the need for future studies to account for and analyze the impact of post-operative systemic therapies on HRQoL outcomes.

### 4.5. Implications for Clinical Practice

The findings of the present review carry significant clinical significance. With heterogeneity in HRQoL following surgery, practitioners must take proactive steps to improve patient recovery at different phases of the post-operative period. Early treatment, such as optimized pain relief, structured psychological treatment, and comprehensive rehabilitation plans, can preclude the post-operative decline in HRQoL and help achieve a more stable recovery process. Furthermore, patient-specific risk factor-guided follow-up treatment has the potential to improve long-term HRQoL outcomes. Significant individual diversity persists even though post-operative HRQoL in HCC patients tends to improve over time. To promote long-term survivability and enhance the general quality of life, post-operative care should incorporate interventions that address the social, psychological, and physical aspects of recovery.

### 4.6. Future Directions

The findings emphasize the need for integrated, multidisciplinary interventions to improve HRQoL in patients with HCC. Given the significant impact of depression, fatigue, and pain, early detection and effective management of these symptoms must be prioritized. Incorporating psychological support, fatigue-reduction strategies, and optimal pain control into routine care may alleviate symptom burden and enhance overall well-being.

Post-operative care should also consider demographic factors such as education, family support, and age, which influence recovery potential and long-term outcomes. Future studies are needed to develop personalized interventions that address both clinical and psychosocial dimensions of HRQoL. Additionally, more research is warranted to assess the sustained effectiveness of such interventions and to establish standardized treatment protocols.

With the increasing adoption of systemic therapies, particularly targeted agents and immunotherapies, future research should also investigate treatment-related toxicities that can substantially impair HRQoL, such as hand-foot syndrome, fatigue, and hypertension. These adverse effects, common among non-surgical patients, demand tailored supportive strategies distinct from those used post-surgically.

Finally, there is a pressing need to harmonize HRQoL assessments across clinical trials. The current heterogeneity in instruments, timing, and domains limits comparability and hinders high-quality meta-analyses. Developing consensus guidelines for HRQoL measurement in trials involving novel therapies and surgical innovations is essential to ensure meaningful, patient-centered outcome evaluation.

### 4.7. Strengths and Limitations

This scoping review provides a comprehensive overview of the literature on HRQoL in HCC patients after surgery, including the factors that influence and the trends that emerge. Having different study designs and measurement tools broadens the range of information gathered. Nevertheless, certain limitations must be mentioned. For instance, the heterogeneity in study populations with respect to HRQoL measurement tools, follow-up periods, and patient populations may lower the generalizability of findings. Moreover, the high degree of heterogeneity in study design, populations, and outcome measures precluded the possibility of conducting a meta-analysis. In addition, the review was limited to English-language research, which may have resulted in the omission of relevant studies published in other languages. Finally, as a scoping review, this study employed a comprehensive but not fully systematic search strategy, which may limit the depth of evidence synthesis compared to a formal systematic review.

## 5. Conclusions

This scoping review highlights that HRQoL among HCC patients worsens postoperatively but improves progressively, varying by type of surgery. Liver resection showed improvement in 3-12 months, while transplantation was associated with long-term benefits irrespective of immunosuppressive problems. Preoperative symptoms, post-operative morbidity, and socioeconomic status were the key determinants of HRQoL. Early interventions like psychological support and symptom management were found to be crucial in ensuring optimal recovery. Subsequent research should focus on standardized HRQoL assessment, individualized interventions, and follow-up outcomes via different surgical methods.

## Supplementary Material

Supplementary Material 1: The PRISMA-ScR checklist; Supplementary Material 2: Search strategy.

## Figures and Tables

**Table 1:** Baseline demographic characteristics of included studies

Author and Year (Country)	Sample size	Age (Mean±SD)	Male n (%)	Aetiology of HCC	Staging of HCC
Tohme et al.,2020 (USA) 18	123	56.5 ± 10.2	85 (69.1%)	Not reported	Not reported
Chiu et al.,2018 (China) 19	332	60.2 ± 10.8	247 (74.4%)	HBV	Stage I-III
Landa et al., 2021 (Germany) 20	81	Not reported	Not reported	Not reported	Early-stage (Milan)
Lei et al., 2016 (China) 21	207 (110 LR, 95 LT)	LR: 45.4 ± 10.0; LT: 48.1 ± 10.2	LR: 92; LT: 83	HBV	Early-stage (Milan)
Chen et al.,2004 (China) 22	81 (34 LR, 47 LT)	LT: 53.2 ± 6.9; LR: 57.8 ± 6.2	LT: 38; LR: 30	HBV & HCV	Not reported
Extraviz et al.,2007 (Spain) 23	90	54.2 ± 10.4	73 (81.1%)	Not reported	Not reported
Xie et al.,2015 (China) 24	102 (58 LR, 44 TACE)	LR: 46.9 ± 8.3; TACE: 44.0 ± 10.6	LR: 48; TACE: 38	HBV	BCLC-B
Ueno et al., 2002 (Japan) 25	96	Not reported	78 (81.3%)	HBV	BCLC-B
Chie et al., 2015 (China) 26	171 (53 LR, 53 ABL, 65 TACE)	LR: 55.1 ± 12.1; ABL: 65.2 ± 11.2; TACE: 64.7 ± 11.5	SRG: 43; ABL: 35; EMB: 53	Not reported	BCLC-A
Mabrouk et al.,2012 (Egypt) 27	122	50.7 ± 9.4	106 (86.9%)	HBV & HCV & alcohol	BCLC-A
Li et al., 2007 (China) 28	161	61.6 ± 12.4	122 (75.8%)	HBV & HCV	Not reported
Mise et al.,2014 (Japan) 29	86	51.4 ± 9.6	77 (89.5%)	Not reported	Early-stage (Milan)
Poon et al.,2001 (USA) 30	66	52.8 ± 11.0	55 (83%)	HBV	pTNM I/II

Abbreviations: HCC: Hepatocellular Carcinoma, HBV: Hepatitis B Virus, HCV: Hepatitis C Virus, BCLC: Barcelona Clinic Liver Cancer, TNM: Tumor, Node, Metastasis, pTNM: Pathological Tumor, Node, Metastasis, LR: Liver Resection, LT: Liver Transplantation, TACE: Transarterial Chemoembolization, Milan: Milan Criteria, ABL: Ablation

**Table 2:** HRQoL trends in patients with HCC post-surgery.

Author and Year (Country)	Type of surgery	HRQoL assessment tool	HRQoL assessment time points	HRQoL trend summary
Tohme et al.,2020 (USA) 18	liver resection	FACT-Hep	4,8,12 months	↓ at 4 months → recovered to baseline at 8 & 12 months
Chiu et al.,2018 (China) 19	liver resection	SF-36	3,6 months	↑ at 3 months → plateau at 6 months
Chen et al.,2004 (China) 22	liver resection	GQLI	2, 5, 10 weeks; 4, 6, 9 months; 1, 1.5, 2 years	↓ early (2-10weeks) → recovered at 4 months→ ↑ beyond baseline in long-term survivors
Extraviz et al.,2007 (Spain) 23	liver transplantation	SF-36	pre-surgery and 6 months post-surgery	significantly ↑ in HRQOL post-surgery
Xie et al.,2015 (China) 24	liver resection & TACE	SF-36	1,3,6,12,24 months	↓ at 1 month (more in resection group) → ↑ at 3-6 months→ resection group better at 1-2 years
Mabrouk et al.,2012 (Egypt) 27	liver transplantation	SF-36	not mentioned	significant ↑ in HRQoL after transplant
Mise et al.,2014 (Japan) 29	liver resection	SF-36	every 3 months post-surgery	Mental HRQoL ↑; Physical ↓ recovered to baseline at 6 months
Poon et al.,2001 (USA) 30	liver resection	FACT-Hep	3,6,9,12,18,24 months	Overall ↑ post-surgery; recurrence leads to ↓ in HRQoL

Abbreviations: HRQoL: Health-Related Quality of Life, FACT-Hep: The Functional Assessment of Cancer Therapy Hepatobiliary, SF-36: The Short Form-36 Health Survey, GQLI: Gastrointestinal Quality of Life Index

**Table 3:** Surgical procedures and follow-up durations of included studies.

Author and Year (Country)	Surgical procedure	Follow-up duration
Tohme et al.,2020 (USA) 18	Liver resection	4, 8, 12 months
Chiu et al.,2018 (China) 19	Liver resection	3, 6 months
Feldbrugge et al.,2021 (Germany) 20	Liver resection & liver transplantation	Median 15 months
Lei et al.,2016 (China) 21	Liver resection & liver transplantation	1-2 months initially, then up to several years
Chen et al.,2004 (China) 22	Liver resection	Pre-surgery to 1.5-2 years post-surgery
Extraviz et al.,2007 (Spain) 23	Liver transplantation	Pre-surgery and 6 months post-surgery
Xie et al.,2015 (China) 24	Liver resection &TACE	1, 3, 6, 12, 24 months
Ueno et al.,2002 (Japan) 25	Liver resection	12 to 60 months
Chie et al.,2015 (China) 26	Liver resection & ablation & TACE	12-15 weeks (liver resection), 4-6 weeks (others)
Mabrouk et al.,2012 (Egypt) 27	Liver transplantation	Not mentioned
Lee et al.,2007 (China) 28	Liver resection	Not mentioned
Mise et al.,2014 (Japan) 29	Liver resection	Every 3 months post-surgery
Poon et al.,2001 (USA) 30	Liver resection	3 to 24 months

Abbreviations: TACE: Transarterial Chemoembolization

**Table 4:** HRQoL assessment tools utilized in the selected studies.

Assessment tool	Domains/Subscales	Scoring system	Studies
SF-36 (n = 7 studies)	Physical FunctioningRole PhysicalBodily PainGeneral HealthVitalitySocial FunctioningRole EmotionalMental Health	Each domain scored from 0 to 100; higher scores indicate better HRQoL. Two summary scores: PCS and MCS	19-21, 23, 24, 27, 29
FACT-Hep (n = 2 studies)	Physical Well-beingSocial/Family Well-beingEmotional Well-beingFunctional Well-beingHepatobiliary Cancer Subscale	Each item was rated on a 5-point Likert scale (0-4); total score ranged from 0-180. Higher scores indicate better HRQoL.	18, 30
GQLI (n = 1 study)	Gastrointestinal SymptomsEmotional Well-beingPhysical StatusSocial FunctioningMedical Treatment Impact	36 items, each scored 1-5; total score range 0-144. Higher scores reflect better HRQoL.	22
WHOQOL-BREF (n = 1 study)	Physical HealthPsychological HealthSocial RelationshipsEnvironment	26 items on a 5-point Likert scale; domain scores transformed to 4-20 or standardized to 0-100. Higher scores indicate better HRQoL.	23
EORTC QLQ-C30 (n = 1 study)	Functional Scales:PhysicalRoleEmotionalCognitiveSocialSymptom Scales:Fatigue, Nausea/Vomiting, Pain, Dyspnea, Insomnia, Appetite loss, Constipation, DiarrheaGlobal Health	All scores standardized to 0-100. Higher scores on functional/global scales = better QoL; higher scores on symptom scales = worse symptoms.	24
Self-designed validated questionnaire (n = 1 study)	Covers physical, psychological, social, and treatment-related aspects	Validated by authors; based on Likert scales. Higher scores are presumed to indicate better HRQoL.	25

Abbreviations: HRQoL: Health-Related Quality of Life, FACT-Hep: The Functional Assessment of Cancer Therapy Hepatobiliary, SF-36: The Short Form-36 Health Survey, GQLI: Gastrointestinal Quality of Life Index, PCS: Physical Component Summary, MCS: Mental Component Summary, EORTC QLQ-C30: European Organisation for Research and Treatment of Cancer Quality of Life Questionnaire-Core 30, WHOQOL-BREF: World Health Organization Quality of Life-BREF, FACT-Hep: Functional Assessment of Cancer Therapy-Hepatobiliary.

**Table 5:** HRQoL Outcomes across Different Surgical Procedures.

Study	Surgical procedure	Short-term HRQoL outcomes	Long-term HRQoL outcomes	Findings
18, 19	Liver resection	Initial decline post-surgery; improvement within 3-12 months	Long-term HRQoL affected by recurrence and complications	Gradual recovery
27	Liver Transplantation	Generally good early recovery	Sustained improvement, but may be impacted by immunosuppression side effects and complications	Higher HRQoL
20	Laparoscopic liver resection	Comparable to open surgery in early outcomes	Not mentioned	Similar HRQoL to transplantation
21	Liver Resection & Transplantation	Both show HRQoL improvement post-treatment	Not differ significantly	Liver resection is possibly better than TACE
26	Liver Resection & TACE	Liver resection is initially more invasive	Potential for higher HRQoL in the surgical group over time	Better outcomes in surgical patients

Abbreviations: HRQoL: Health-Related Quality of Life, TACE: Transarterial Chemoembolization

**Table 6:** Key factors influencing HRQoL outcomes of the selected studies

Study	Factor type	Specific factors	Findings
18	Preoperative	Depressive symptoms, pain, fatigue	Strong contributors to low preoperative HRQoL
18	Post-operative	Tumor recurrence, tumor characteristics, persistent symptoms	Major influences on long-term HRQoL
19, 28	Sociodemographic	Age, gender, education level, family and social support	Influenced both short- and long-term HRQoL outcomes
20, 22, 29	Surgical	Extent of surgery, surgical complications, perioperative outcomes	Larger resections and complications linked to poorer HRQoL outcomes
24, 25, 30	Disease recurrence	HCC recurrence	A strong predictor of HRQoL decline over time

Abbreviations: HRQoL: Health-Related Quality of Life, HCC: Hepatocellular Carcinoma

**Table 7:** Changes in HRQoL over time

Study	Surgical method	HRQoL changes over time	Key observations
29	Liver resection	Decline at 3 months; recovery by 6 months	Physical health reached the lowest point at 3 months; returned to baseline by 6 months; mental health improved.
19	Liver resection	Gradual improvement sustained over time	Significant HRQoL gains post-surgery, especially in non-recurrent cases.
30	Liver resection	Sustained improvement in multiple domains	Long-term benefits were noted if no recurrence occurred.
28	Liver transplantation	Improvement post-surgery, but HRQoL below population norms	HRQoL significantly improved, though it did not reach levels of the general population in long-term follow-up.

Abbreviations: HRQoL: Health-Related Quality of Life

## Abbreviations

ABL: Ablation
BCLC: Barcelona Clinic Liver Cancer
EORTC QLQ-C30: European Organisation for Research and Treatment of Cancer Quality of Life Questionnaire Core 30
EQ-5D: EuroQol 5-Dimensions
ERAS: Enhanced Recovery After Surgery
FACT-Hep: Functional Assessment of Cancer Therapy-Hepatobiliary
FACT-HS: Functional Assessment of Cancer Therapy-Hepatocellular Symptoms
FHSI-8: Functional Assessment of Cancer Therapy-Hepatobiliary Symptom Index-8GQLI
GQLI: Gastrointestinal Quality of Life Index
FACT-Hep: Functional Assessment of Cancer Therapy-Hepatobiliary
HBV: Hepatitis B Virus
HCC: Hepatocellular Carcinoma
HCV: Hepatitis C Virus
HRQoL: Health-Related Quality of Life
JBI: Joanna Briggs Institute
LR: Liver Resection
LT: Liver Transplantation
Milan: Milan Criteria
PRISMA-ScR: Preferred Reporting Items for Systematic Reviews and Meta-Analyses extension for Scoping Reviews
QLQ-HCC18: European Organisation for Research and Treatment of Cancer Quality of Life Questionnaire-Hepatocellular Carcinoma Module
SF-36: 36-Item Short Form Survey
TACE: Transarterial Chemoembolization
