# Supplementary material for: Health-Related Quality of Life in Patients with Hepatocellular Carcinoma Post-Surgery: A Scoping Review
Source: Int J Med Sci. 2025 Jul 28;22(14):3709–21. doi: 10.7150/ijms.115946 (PMC12434815; doi:10.7150/ijms.115946)

Search Strategy

Pubmed: 355

(("liver cancer"[Title/Abstract] OR "hepatocellular carcinoma"[Title/Abstract] OR "hepatocarcinoma"[Title/Abstract] OR "HCC"[Title/Abstract])
AND "quality of life"[Title/Abstract]
AND (postoperat*[Title/Abstract] OR post-operat*[Title/Abstract] OR postsurg*[Title/Abstract] OR post-surg*[Title/Abstract] OR surg*[Title/Abstract] OR operat*[Title/Abstract]))

Search: **(((((liver cancer[Title/Abstract]) OR (hepatocellular carcinoma[Title/Abstract])) OR (hepatocarcinoma[Title/Abstract])) OR (HCC[Title/Abstract])) AND (quality of life[Title/Abstract])) AND ((((((postoperat*[Title/Abstract]) OR (post-operat*[Title/Abstract])) OR (postsurg*[Title/Abstract])) OR (post-surg*[Title/Abstract])) OR (surg*[Title/Abstract])) OR (operat*[Title/Abstract]))**

Scopus: 439

TITLE-ABS("liver cancer" OR "hepatocellular carcinoma" OR "hepatocarcinoma" OR "HCC")
AND TITLE-ABS("quality of life")
AND TITLE-ABS(postoperat* OR post-operat* OR postsurg* OR post-surg* OR surg* OR operat*)

TITLE-ABS("liver cancer" OR "hepatocellular carcinoma" OR "hepatocarcinoma" OR "HCC") AND TITLE-ABS("quality of life") AND TITLE-ABS("postoperat*" OR "post-operat*" OR "postsurg*" OR "post-surg*" OR "surg*" OR "operat*")

Web of science: 481

TS=("liver cancer" OR "hepatocellular carcinoma" OR "hepatocarcinoma" OR "HCC")
AND TS=("quality of life")
AND TS=(postoperat* OR post-operat* OR postsurg* OR post-surg* OR surg* OR operat*)

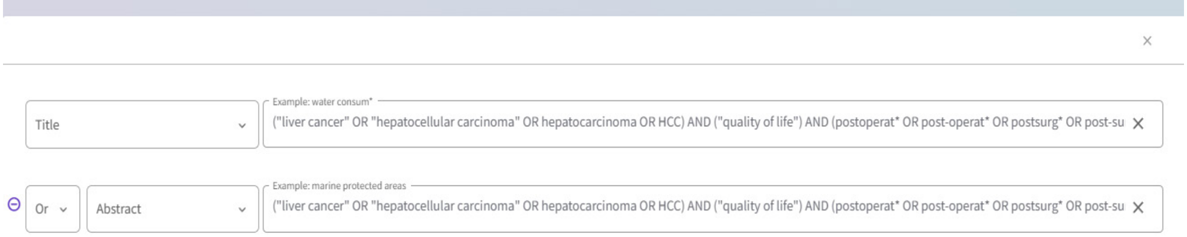

Supplement: Supplementary file 1 — Supplementary Material 1: The PRISMA-ScR checklist; Supplementary Material 2: Search strategy. [file ijmsv22p3709s1.pdf]
